# Protein Adsorption to Titanium and Zirconia Using a Quartz Crystal Microbalance Method

**DOI:** 10.1155/2017/1521593

**Published:** 2017-01-29

**Authors:** You Kusakawa, Eiji Yoshida, Tohru Hayakawa

**Affiliations:** Department of Dental Engineering, Tsurumi University School of Dental Medicine, 2-1-3 Tsurumi, Tsurumi-ku, Yokohama 230-8501, Japan

## Abstract

Protein adsorption onto titanium (Ti) or zirconia (ZrO_2_) was evaluated using a 27 MHz quartz crystal microbalance (QCM). As proteins, fibronectin (Fn), a cell adhesive protein, and albumin (Alb), a cell adhesion-inhibiting protein, were evaluated. The Ti and ZrO_2_ sensors for QCM were characterized by atomic force microscopy and electron probe microanalysis observation, measurement of contact angle against water, and surface roughness. The amounts of Fn and Alb adsorbed onto the Ti and ZrO_2_ sensors and apparent reaction rate were obtained using QCM measurements. Ti sensor showed greater adsorption of Fn and Alb than the ZrO_2_ sensor. In addition, amount of Fn adsorbed onto the Ti or ZrO_2_ sensors was higher than that of Alb. The surface roughness and hydrophilicity of Ti or ZrO_2_ may influence the adsorption of Fn or Alb. With regard to the adsorption rate, Alb adsorbed more rapidly than Fn onto Ti. Comparing Ti and ZrO_2_, Alb adsorption rate to Ti was faster than that to ZrO_2_. Fn adsorption will be effective for cell activities, but Alb adsorption will not. QCM method could simulate* in vivo* Fn and Alb adsorption to Ti or ZrO_2_.

## 1. Introduction

Titanium (Ti) has been widely used as dental implant material because of its superior mechanical properties and biocompatibility [1]. The tight and direct bonding of Ti is known as osseointegration [2]. Recently high-strength partially stabilized zirconia (ZrO_2_) implants have attracted attention as an alternative to Ti implants [3–6]. Partially stabilized ZrO_2_ is yttria-stabilized tetragonal zirconia polycrystal and has superior mechanical properties such as high fracture toughness and better esthetic performance.

When implant materials are inserted into bone tissue, the adsorption of body-fluid proteins, including extracellular matrix components, at the implant-tissue interface is the first biological response to the implants [7–9]. Trindade et al. insisted that adsorption of proteins on implant surface is the first step of the path to osseointegration [10]. After protein adsorption, some biological events such as immune responses and macrophage differentiation occurred. Afterwards, bone formation started at the implant interface.

Surface modification by cell adhesive proteins such as fibronectin or collagen was reported to promote tissue healing and remodeling process. For example, fibronectin coating on titanium promoted osteoblastic attachment differentiation and implant osseointegration [11–13]. Our previous studies revealed that fibronectin immobilization onto titanium altered gene expression and the expression of some genes related to mineralization process were upregulated [14].

On the contrary, albumin is a major component in the human saliva and is known as cell adhesion-inhibiting protein. Kawashita et al. reported that albumin adsorbed hydroxyapatite and alumina inhibited the adhesion and spreading of MC3T3-E1 cells [15]. Thus, it is important to observe protein adsorption to Ti and ZrO_2_ to identify biological properties of Ti and ZrO_2_.

There are several methods for analyzing protein adsorption such as infrared reflection spectroscopy, ellipsometry, and surface plasmon resonance [16]. Among them, quartz crystal microbalance (QCM) technique is straightforward method for detecting the adsorption of proteins onto a material surface by measuring differences in the oscillating frequency of the quartz cell [17]. The adsorption of protein onto the surface of the oscillating quartz crystal causes the oscillation frequency to decrease in relation to the amount of protein bound to the crystal surface.

In the present study, we aimed to investigate the protein adsorption onto Ti and ZrO_2_ using a 27 MHz QCM, which enabled measurements with high sensitivity and low noise [18, 19]. Fn and Alb were both components in saliva. As mentioned above, Fn is a cell adhesive protein and is known to play a crucial role in adhesion-dependent cellular activities including attachment, proliferation, and differentiation [20]. On the other hand, Alb is cell adhesion-inhibiting protein which is opposite to Fn. Thus, Fn and Alb were evaluated as test proteins.

Null hypothesis tested was that the difference of materials, Ti and ZrO_2_, and that of proteins, Fn and Alb, did not influence the protein adsorption behaviors.

## 2. Materials and Methods

### 2.1. QCM Apparatus and Sensors

**Figure 1:**
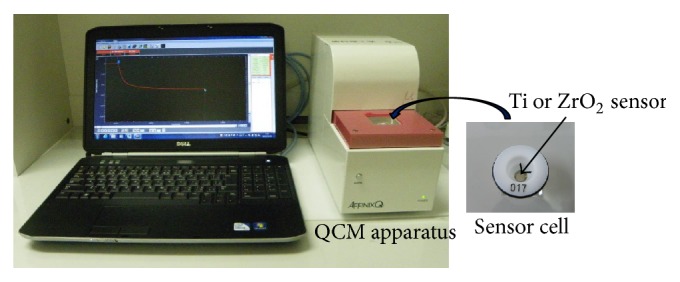
Appearance of 27 MHz QCM apparatus.

A 27 MHz QCM (AT cut shear mode, AFFINIX QN*μ*, ULVAC, Inc., Kanagawa, Japan) was used. As shown in Figure 1, Ti or ZrO_2_ sensor was assembled in a sensor cell with a volume of 550 *μ*L. Then sensor cell with Ti or ZrO_2_ sensor was mounted in the cell socket of the QCM apparatus. A temperature control system and stirring bar were installed, with the temperature maintained at 25 ± 1°C, and the solution in the cell was stirred during measurements.

Ti and ZrO_2_ sensors were used. The Ti and ZrO_2_ sensors were prepared by sputter coating of each material on a gold electrode. The deposition conditions for the Ti and ZrO_2_ sensors are shown in Table 1. Ti or ZrO_2_ disks (Quartz 4N, ULVAC, Inc., Kanagawa, Japan) were used as a target, and the deposition of each material was performed using sputtering deposition equipment (CS200, ULVAC, Inc., Kanagawa, Japan). Ti sputtering was performed in argon gas, and ZrO_2_ sputtering was done in oxygen gas. Each sensor was irradiated with ultraviolet radiation (BioForce Nanosciences Holdings Inc., US) for 20 minutes before QCM measurement.

### 2.2. Atomic Force Microscope Observation of Ti and ZrO_2_ Sensors before QCM Protein Adsorption

An atomic force microscope (AFM; Nanosurf Easyscan 2, Nanosurf, AG, Switzerland) observation identified the surface condition and surface roughness of the Ti and ZrO_2_ sensors. AFM images were captured in air. Tapping mode silicon probes (Tap190AL-G, force contact 48 N/m Budget Sensors, Bulgaria) with resonance frequencies of approximately 190 kHz were used for imaging. AFM images were obtained for an area of 2 × 2 *μ*m^2^.

### 2.3. Electron Probe Microanalysis of Ti and ZrO_2_ Sensors

The Ti and ZrO_2_ sensor surfaces after ultraviolet irradiation were evaluated by electron probe microanalysis (EPMA; JXS-8900RL, JEOL Ltd., Tokyo, Japan) at an accelerating voltage of 15 kV by detecting the X-ray intensity of Ti-K*α*, Zr-K*α*. Ti or Zr surface mapping was performed.

### 2.4. Contact Angle Measurements of Ti and ZrO_2_ Sensors

The contact angles of the Ti and ZrO_2_ sensor surfaces with respect to double-distilled water were measured using a contact angle meter (DMe-201, Kyowa Interface Science Co. Ltd., Tokyo, Japan) after ultraviolet irradiation of each sensor. The water drop volume was maintained at 0.5 *μ*L, and three measurements of 10 seconds each were made for each surface type. Measurements were performed at the same room temperature (25 ± 1°C) and humidity (45 ± 1%).

### 2.5. QCM Measurements of Albumin and Fibronectin

Human plasma fibronectin (Fn, Harbor Bio-Products, MA, USA) or bovine serum albumin (Alb, Wako Pure Chemical Industries, Ltd., Japan) was dissolved in a phosphate-buffered saline (PBS) solution (pH 7.4) at a concentration of 0.5 mg/mL.

**Figure 2:**
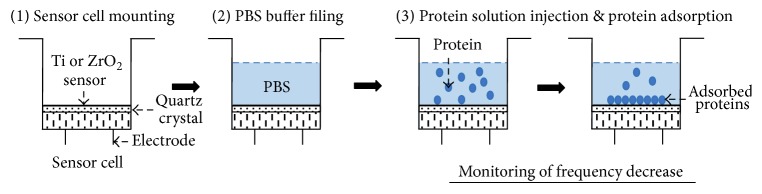
The procedure for QCM measurements. All measurements were performed using a 27 MHz QCM.

The procedure for QCM measurement is illustrated in Figure 2. The sensor cell with a Ti or ZrO_2_ sensor was mounted in the QCM apparatus. Then, 500 *μ*L PBS was filled into the sensor cell. Afterwards, 5 *μ*L of protein solution was injected into the PBS solution in the sensor cell. Protein adsorption onto the Ti or ZrO_2_ sensors caused the frequency decrease. The frequency decrease was monitored until 30 min after protein injection. The amount of protein adsorbed onto each surface (Δ*m*) at 30 min after protein injection was calculated using Sauerbrey's equation [21]. According to the equation, a frequency decrease of 1 Hz corresponds to 0.61 ± 0.1 ng/cm^2^ adsorption on the sensor in this 27 MHz QCM system. By curve-fitting for the Δ*F* curve against the adsorption time, the apparent reaction rate, *K*obs, in the following equation was obtained. Δ*F*_*∞*_ is the frequency shift at infinite time.

(1) $$\begin{array}{c} \Delta F_{t}=\Delta F_{\infty }\left(\;1-e^{-K\text{obs}\cdot t}\;\right). \end{array}$$

One measurement used one Ti or ZrO_2_ sensor. Three runs of QCM measurements were performed during 30 min. Thus there are three Ti or ZrO_2_ sensors for Fn adsorption and three Ti or ZrO_2_ sensors for Alb adsorption. After protein adsorption, AFM images of the Ti and ZrO_2_ sensors were also observed on the conditions same as the above.

### 2.6. Statistical Analyses

Significant differences were determined using statistical analysis software (GraphPad Prism, GraphPad Software Inc., San Diego, CA, USA). Statistical significance was set at *p* < 0.05. Nonpaired *t*-test was employed to compare data obtained from contact angle measurements, in surface roughness measurements, and in QCM measurements. The adsorbed amounts and *K*obs were compared between Ti and ZrO_2_ sensors for each protein and between Fn and Alb for each sensor.

## 3. Results

### 3.1. Characterization of the Ti and ZrO_2_ Sensors

**Figure 3:**
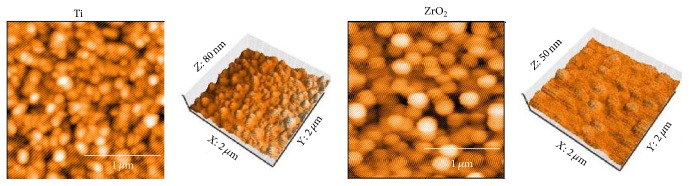
AFM images of Ti and ZrO_2_ QCM sensors before protein adsorption. All measurements were performed in tapping mode using aluminum reflex coating silicon long cantilever with a resonance frequency of approximately 190 kHz and force contact of 48 N/m. AFM images were obtained for an area of 2 × 2 *μ*m^2^.

**Figure 4:**
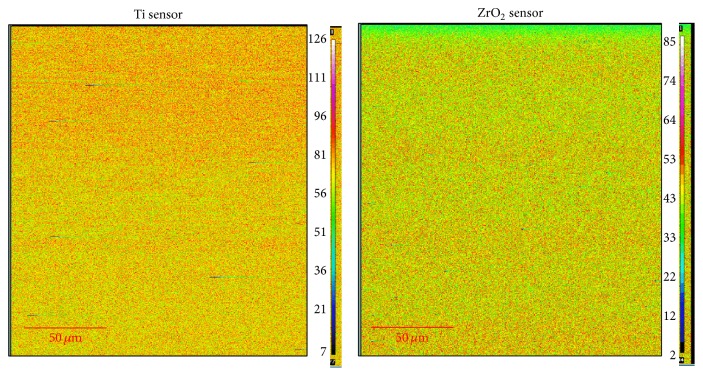
EPMA mapping images of Ti and ZrO_2_ QCM sensors. All measurements were performed at an accelerating voltage of 15 kV.

Contact angle and surface roughness of the Ti and ZrO_2_ sensors are listed in Table 2. There was significant difference in contact angle (*p* < 0.05). Surface roughness values indicated that the Ti surface was significantly rougher than that for ZrO_2_ (*p* < 0.05). AFM images of the Ti and ZrO_2_ sensors are shown in Figure 3. Spherical particles with a diameter of 0.2–0.3 *μ*m were observed on both surfaces, and more bumps were recognized on the Ti sensor surface compared with the ZrO_2_ surface.  Figure 4 shows the elementary distribution of Ti and Zr on each sensor surface by EPMA analysis. Homogeneous sputter coating of Ti and Zr on each sensor surface was confirmed.

### 3.2. QCM Measurements

**Figure 5:**
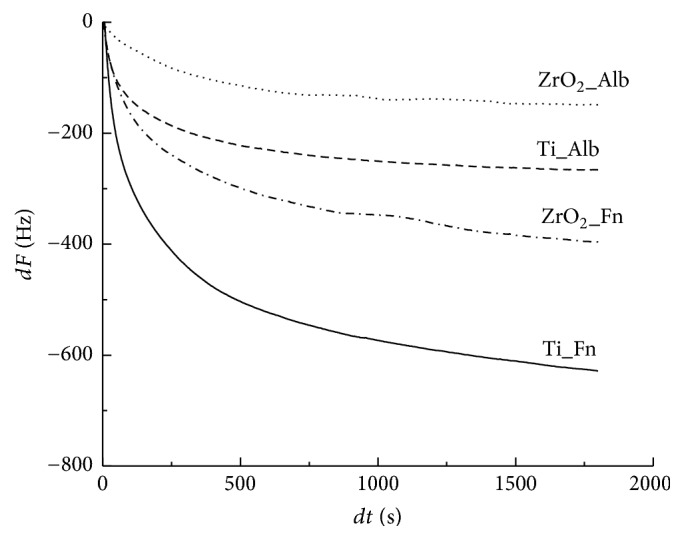
Frequency shift for Fn or Alb adsorption onto Ti or ZrO_2_ sensors using QCM measurements.

**Figure 6:**
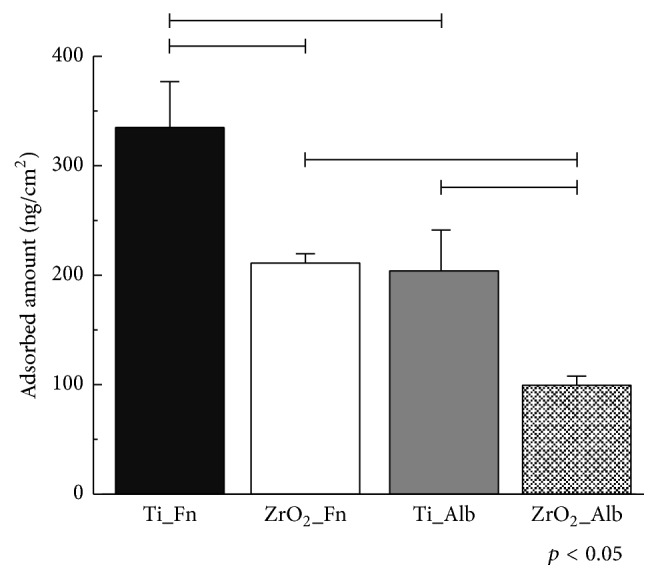
Adsorbed amount of Fn or Alb onto Ti or ZrO_2_ sensors. Connected bar: significant difference (*p* < 0.05).

**Figure 7:**
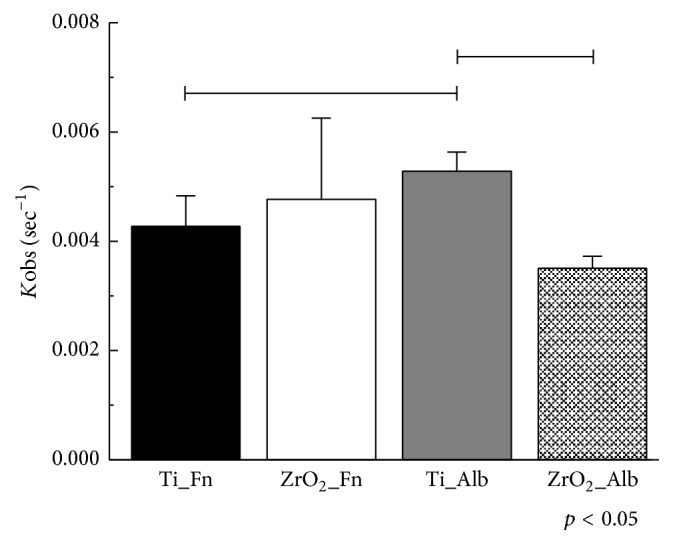
*K*obs values of Fn or Alb adsorption to Ti or ZrO_2_ sensors by nonlinear fitting analysis. Connected bar: significant difference (*p* < 0.05).

Frequency decrease was observed immediately after the injection of protein solution into both Ti and ZrO_2_ sensors as shown in Figure 5. A greater degree of frequency decrease corresponds to a greater degree of adsorption of the protein to each sensor. Comparing the Ti and ZrO_2_ sensors, Ti exhibited a more rapid and greater degree of decrease in frequency. Fn showed a greater degree of frequency decrease than Alb onto both the Ti and ZrO_2_ sensors.  Figure 6 shows the adsorbed amounts at 30 min after the injection of each protein calculated using Sauerbrey's equation [22]. Comparing the two sensors, the amounts of Fn or Alb adsorbed onto Ti were significantly higher than those onto ZrO_2_ (*p* < 0.05). Significantly greater amounts of Fn adsorbed onto the Ti and ZrO_2_ sensors than Alb onto the respective sensor (*p* < 0.05). *K*obs values of Fn and Alb are shown in Figure 7. A larger value of *K*obs indicated a more rapid reaction rate. No significant difference was observed between Fn adsorption onto Ti or ZrO_2_ (*p* > 0.05), but for Alb adsorption there was a significant difference in *K*obs between Ti and ZrO_2_ (*p* < 0.05). Comparing between Fn and Alb, there was a significant difference in *K*obs (*p* < 0.05) for Ti but not for ZrO_2_ (*p* > 0.05).

**Figure 8:**
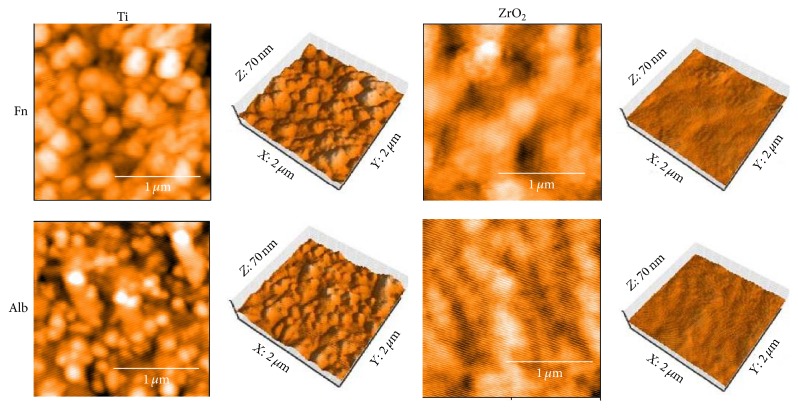
AFM images of Ti and ZrO_2_ QCM sensors after protein adsorption. All measurements were performed in tapping mode using aluminum reflex coating silicon long cantilever with a resonance frequency of approximately 190 kHz and force contact of 48 N/m. AFM images were obtained for an area of 2 × 2 *μ*m^2^.

AFM images of the Ti and ZrO_2_ sensors after protein immobilization are shown in Figure 8, and surface roughness values are listed in Table 3. Both surfaces were covered with adsorbed Fn or Alb. The surface of ZrO_2_ had larger protein globules, and a more flattened surface was recognized after protein adsorption. The Ti surface was still significantly rougher than the ZrO_2_ surface after protein adsorption (*p* < 0.05). The surfaces of Ti or ZrO_2_ were significantly rougher after Fn absorption than after Alb adsorption (*p* < 0.05). Surface roughness was significantly decreased by protein adsorption (*p* < 0.05) except for Fn adsorption onto Ti.

## 4. Discussion

In the present study, we evaluated the adsorption of Fn and Alb onto Ti or ZrO_2_ surfaces using the QCM method. It revealed that the difference of materials, Ti and ZrO_2_, and that of proteins, Fn and Alb, influenced the protein adsorption behaviors. Therefore, the null hypothesis was rejected.

Generally, the adsorption of proteins onto biomaterials is controlled by many factors such as electrostatic and ionic interaction, hydrogen bond or chemical bond formation, and hydrophobic-hydrophilic interaction. It was predicted that electrostatic interactions between protein and Ti were dominant in protein adsorption [23]. The zeta potentials of Ti and ZrO_2_ at pH 7.4 were reported to be approximately −87 and −40 mV, respectively [24]. This means that both the Ti and ZrO_2_ surfaces are negatively charged and Ti is more negatively charged than ZrO_2_. Yoshida and Hayakawa found that greater amounts of positively charged lactoferrin adsorbed onto more negatively charged Ti at pH 7.4 than onto ZrO_2_ [24]. The isoelectric points of Fn and Alb are approximately 5-6 and 4.7–4.9, respectively [22, 25]. Fn and Alb were negatively charged in the present buffer conditions at pH 7.4. Thus, electrostatic repulsion occurred between each protein, Fn or Alb, and each sensor, Ti or ZrO_2_. The more negatively charged Ti material should be associated with lower adsorption levels than ZrO_2_.

However, the present results showed the opposite, with greater amounts of Fn or Alb adsorbed onto more negatively charged Ti. Yan et al. investigated Alb adsorption onto CoCrMo alloy at different pH values [26]. At pH 10.0, Alb was negatively charged and the alloy surface was positively charged. The electrostatic attraction between Alb and the alloy surface should enhance adsorption. However, they found that maximum adsorption occurred at the isoelectric point of Alb, pH 4.7, and speculated that the repulsion of charged Alb molecules and conformational changes in Alb molecules influenced the adsorption behavior. It was suggested that repulsion molecules and/or conformational changes of Fn and Alb in the present conditions may provide the opposite results with previous results. More detailed studies testing adsorption at different buffer pH values or with different concentrations of proteins will elucidate the reason for this opposite result.

Surface roughness of the material surface also influences protein adsorption, and more proteins adsorbed onto a rougher surface [27]. Ti used in this study had a rougher surface than ZrO_2_ at the nanoscale level. In the present conditions, the rougher Ti surface could enhance the adsorption of Fn and Alb.

Comparing Fn and Alb, greater amounts of Fn adsorbed onto both the Ti and ZrO_2_ surfaces. Wei et al. evaluated the adsorption of Fn and Alb to surfaces with different wettability [28]. They found that Fn showed higher levels of adsorption onto hydrophilic and hydrophobic surfaces with contact angles of around 0° and 80° and Alb adsorbed onto a hydrophobic surface with a contact angle of around 80°. The present Ti and ZrO_2_ surfaces were both hydrophilic. Thus, adsorption of Fn progressed but that of Alb was suppressed on the hydrophilic Ti and ZrO_2_ surfaces.

Fn is known as cell adhesion protein and Alb is cell adhesion-inhibiting protein. It is presumed that cell responses such as attachment and differentiations will be enhanced by Fn adsorption, but not by Alb adsorption [20]. *K*obs measurements indicated that Alb was adsorbed more rapidly than Fn to Ti. Moreover, comparing Ti and ZrO_2_, Alb adsorption onto Ti was more rapid than that onto ZrO_2_. It was presumed that Alb adsorbs earlier onto Ti than Fn at an early stage of adsorption and afterwards Fn adsorption gradually becomes dominant on Ti. For ZrO_2_, the amount of Fn adsorbed was less than that onto Ti, but the slower adsorption rate of Alb will be benefit for cell responses.

Present QCM study could simulate* in vivo* Fn and Alb adsorption to Ti or ZrO_2_ when implant material will be implanted in the bone tissue but not directly demonstrate the progression of osseointegration. The situation* in vivo* is more complex, and various kinds of protein may participate in adsorption. Mishima et al. investigated the adsorption properties of the cytokine CXCL12, which is expressed during bone healing and osseointegration, to a surface composed of modified Ti using the QCM method and found that superhydrophilic surfaces increased the adsorption of CXCL12 [29]. Interactions among proteins will also influence tissue behaviors. More detailed studies on protein adsorption with other kinds of proteins and examining competition among protein adsorption will be needed.

## 5. Conclusions

The present study revealed the basic adsorption behaviors of Fn and Alb onto Ti and ZrO_2_ using a QCM method. Greater amounts of Fn and Alb were absorbed onto Ti than onto ZrO_2_. The amounts of Fn absorbed onto Ti or ZrO_2_ were higher than that of Alb. With regard to the adsorption rate, Alb adsorbed more rapidly than Fn onto Ti. Comparing Ti and ZrO_2_, the Alb adsorption rate onto Ti was more rapid than that onto ZrO_2_. QCM method could simulate* in vivo* Fn and Alb adsorption to Ti or ZrO_2_.

## Figures and Tables

**Table 1:** Depositions conditions of the Ti and ZrO_2_ sensors.

Sensor	Target	Atmospheric gas	Pressure (Pa)	Sputtering time (min)

Ti	99.99% pure Ti	Argon	0.2	30
ZrO_2_	Zirconium	Oxygen	0.5	30

**Table 2:** Contact angle and surface roughness of Ti and ZrO_2_ sensors before protein adsorption (mean ± SD, *n* = 3).

Sensor	Contact angle (°)	Surface roughness (nm/4 *µ*m^2^)
Ti	4.9 ± 0.8^a^	4.45 ± 0.55^c^
ZrO_2_	7.3 ± 2.2^b^	2.76 ± 0.08^d^

Cells with different letters denote significant differences between Ti and ZrO_2_ (*p* < 0.05).

**Table 3:** Surface roughness of Ti and ZrO_2_ sensors after protein adsorption (nm/4 *µ*m^2^) (mean ± SD, *n* = 3).

Sensor	Fn	Alb
Ti	3.96 ± 0.25^a,A^	3.25 ± 0.31^c,B^
ZrO_2_	1.24 ± 0.06^b,C^	1.07 ± 0.08^d,D^

Cells with different letter denote significant differences (*p* < 0.05), with lowercase letters for Ti versus ZrO_2_ for the same protein and uppercase letters for Fn versus Alb for the same sensor.

## References

[1] Ananth H., Kundapur V., Mohammed H. S., Anand M., Amarnath G. S., Mankar S. (2015). A review on biomaterials in dental implantology. *International Journal of Biomedical Science*.

[2] Brånemark P.-I. (1983). Osseointegration and its experimental background. *The Journal of Prosthetic Dentistry*.

[3] Wenz H. J., Bartsch J., Wolfart S., Kern M. (2008). Osseointegration and clinical success of zirconia dental implants: a systematic review. *International Journal of Prosthodontics*.

[4] Andreiotelli M., Wenz H. J., Kohal R.-J. (2009). Are ceramic implants a viable alternative to titanium implants? A systematic literature review. *Clinical Oral Implants Research*.

[5] Apratim A., Eachempati P., Krishnappa Salian K., Singh V., Chhabra S., Shah S. (2015). Zirconia in dental implantology: a review. *Journal of International Society of Preventive and Community Dentistry*.

[6] Hashim D., Cionca N., Courvoisier D. S., Mombelli A. (2016). A systematic review of the clinical survival of zirconia implants. *Clinical Oral Investigations*.

[7] Horbett T. A. (1993). Chapter 13 principles underlying the role of adsorbed plasma proteins in blood interactions with foreign materials. *Cardiovascular Pathology*.

[8] Wälivaara B., Aronsson B.-O., Rodahl M., Lausmaa J., Tengvall P. (1994). Titanium with different oxides: in vitro studies of protein adsorption and contact activation. *Biomaterials*.

[9] Puleo D. A., Nanci A. (1999). Understanding and controlling the bone-implant interface. *Biomaterials*.

[10] Trindade R., Albrektsson T., Tengvall P., Wennerberg A. (2016). Foreign body reaction to biomaterials: on mechanisms for buildup and breakdown of osseointegration. *Clinical Implant Dentistry and Related Research*.

[11] Bhola R., Su F., Krull C. E. (2011). Functionalization of titanium based metallic biomaterials for implant applications. *Journal of Materials Science: Materials in Medicine*.

[12] Bierbaum S., Hempel U., Geissler U. (2003). Modification of Ti6AL4V surfaces using collagen I, III, and fibronectin. II. Influence on osteoblast responses. *Journal of Biomedical Materials Research Part A*.

[13] Petrie T. A., Reyes C. D., Burns K. L., García A. J. (2009). Simple application of fibronectin-mimetic coating enhances osseointegration of titanium implants. *Journal of Cellular and Molecular Medicine*.

[14] Pugdee K., Shibata Y., Yamamichi N. (2007). Gene expression of MC3T3-E1 Cells on fibronectin-immobilized titanium using tresyl chloride activation technique. *Dental Materials Journal*.

[15] Kawashita M., Hayashi J., Kudo T.-A. (2014). MC3T3-E1 and RAW264.7 cell response to hydroxyapatite and alpha-type alumina adsorbed with bovine serum albumin. *Journal of Biomedical Materials Research Part A*.

[16] Martins M. C., Sousa S. R., Antunes J. C., Barbosa M. A. (2011). Protein adsorption characterization. *Nanotechnology in Regenerative Medicine*.

[17] Murray B. S., Deshaires C. (2000). Monitoring protein fouling of metal surfaces via a quartz crystal microbalance. *Journal of Colloid and Interface Science*.

[18] Furusawa H., Takano H., Okahata Y. (2008). Transient kinetic studies of pH-dependent hydrolyses by exo-type carboxypeptidase P on a 27-MHz quartz crystal microbalance. *Analytical Chemistry*.

[19] Yoshimine H., Kojima T., Furusawa H., Okahata Y. (2011). Small mass-change detectable quartz crystal microbalance and its application to enzymatic one-base elongation on DNA. *Analytical Chemistry*.

[20] Sakai T., Johnson K. J., Murozono M. (2001). Plasma fibronectin supports neuronal survival and reduces brain injury following transient focal cerebral ischemia but is not essential for skin-wound healing and hemostasis. *Nature Medicine*.

[21] Sauerbrey G. (1959). Verwendung von Schwingquarzen zur Wägung dünner Schichten und zur Mikrowägung. *Zeitschrift für Physik*.

[22] Mori O., Imae T. (1997). AFM investigation of the adsorption process of bovine serum albumin on mica. *Colloids and Surfaces B: Biointerfaces*.

[23] Yoshida E., Hayakawa T. (2013). Adsorption study of pellicle proteins to gold, silica and titanium by quartz crystal microbalance method. *Dental Materials Journal*.

[24] Yoshida E., Hayakawa T. (2016). Adsorption analysis of lactoferrin to titanium, stainless steel, zirconia, and polymethyl methacrylate using the quartz crystal microbalance method. *BioMed Research International*.

[25] Boughton B. J., Simpson A. W. (1984). The biochemical and functional heterogeneity of circulating human plasma fibronectin. *Biochemical and Biophysical Research Communications*.

[26] Yan Y., Yang H., Su Y., Qiao L. (2015). Albumin adsorption on CoCrMo alloy surfaces. *Scientific Reports*.

[27] Hannig M., Joiner A. (2006). The structure, function and properties of the acquired pellicle. *Monographs in Oral Science*.

[28] Wei J., Igarashi T., Okumori N. (2009). Influence of surface wettability on competitive protein adsorption and initial attachment of osteoblasts. *Biomedical Materials*.

[29] Mishima N., Iida M., Nakayama N., Hayakawa T., Yoshinari M. (2016). Adsorption property of cytokine CXCL12 to surface-modified titanium with superhydrophilicity. *Journal of Japanese Society of Oral Implantology*.

